# Foreigners welcome? Categorizing change in German mass media discourse with Latent Semantic Analysis (LSA)

**DOI:** 10.1371/journal.pone.0340164

**Published:** 2026-02-13

**Authors:** Arianna Haviv Zehner, Marco Fölsch, Natalja Menold, Manuel Holz, Britta Maskow, Jochen Mayerl

**Affiliations:** 1 Institute for Sociology, Dresden University of Technology, Dresden, Germany; 2 Institute for Sociology, Chemnitz University of Technology, Chemnitz, Germany; National University of Malaysia Faculty of Education: Universiti Kebangsaan Malaysia Fakulti Pendidikan, MALAYSIA

## Abstract

Mass media is often investigated for its influence on public opinion. However, media analysis often relies on measuring term prevalence, elements of framing, and determining bias. New approaches to media analysis are advantageous to the social sciences. Leveraging the German General Social Survey (GGSS), we utilize Latent Semantic Analysis (LSA) to categorize and compare discourse for key points of time (2006, 2010, 2012, 2016, 2021), with over 10,000 media articles from several German media outlets. We focus on the migration and integration of foreigners in Germany and the competing discourse narratives surrounding these events. We adapt the term “foreigner” (Ausländer) in media text; German compound variations such as Ausländerproblem (foreigner problem) and Ausländerintegration (foreigner integration) are central to the discourse analysis. Based on semantic meaning and co-occurrence, these compound terms are grouped into four categories: Administration and Policy, Social Integration, Xenophobia, and Limiting Migration. Results demonstrate that Social Integration discourse becomes more prevalent over time. A subsequent sentiment analysis reveals that Social Integration discourse is not positive but neutral – other categories reflect a negative bias. We therefore discuss computational applications for the enhancement of media analysis, as well as challenges to contextualizing survey data.

## Introduction

Mass media refers to any form of communication that simultaneously reaches a wide audience, including radio, TV, newspapers, magazines, and the internet [1]. Studying which topics are reported in mass media, with which details, connotations, and framing is crucial in understanding mass media discourse trends. Media discourse can uncover a great deal about society, including the public support of political and military conflicts [2], opinions on economic and environmental issues [3], and European matters [4]. Ever since World War I, there has been an interest in media discourse and the potential influence media may have on consumers. Media influence specifically refers to the effect media has on shaping public opinion and/or behavior. This research began first with investigating the effects of propaganda in media during World War I [5] and has long since been extended into various other topics, including immigration and integration [6,7].

Immigration is defined as the movement or change of residence by an individual or groups of people [8]. However, more specific definitions assert that the true marker of immigration is the change of social setting [9]: therefore, immigration marks a new social life in a new place. Social integration is defined as the opposite of social isolation (i.e., when someone establishes social and/or institutional connections, or community participation) [10].

International events such as migration and the subsequent process of integration often happen as abstract phenomena. These phenomena may not be directly experienced on an individual level, but mass media provides information superseding personal experience [11,12], thus playing a key role in the way we perceive world events.

Migration and social integration have ultimately changed the German sociopolitical climate over time [13]. Germany remains one of the top destinations for immigration in Europe [14], as such, there is historically grounded context surrounding themes of immigration and integration in Germany:

Post World War II, Germany was an ethnically homogenous country, divided into East and West factions. West Germany, although homogenous, was more open to the world than its Eastern counterpart and relied on migrant workers for much of the 1950s and 1960s. These workers came predominantly from Turkey, Italy, and Spain [15]. However, these migrants were accepted under the stipulation that they would eventually leave; the increasing working migrant population began to worry the ethnically homogenous host country. As a result, there was a recruitment ban in 1972 [16]. Workers which had already been living and working in Germany for several years (if not decades), did not return to their places of origin, but rather had sent for their families to join them in Germany. Ultimately (and perhaps ironically), this resulted in an increasing foreign population [16]. Resentment over the inadvertent rise had poured into the following decades; the Kohl Government (1982-1998) famously quoted “We are not a country of immigration” [17].

In parallel to these sociopolitical developments, key terminology within discourse had been used to refer to migrants. The most controversial example is the term Ausländer (foreigner), which has developed a negative connotation over time, due to various use-cases, as seen in housing discrimination, in which housing offices advertised “no foreigners (Ausländer)” [18]. Similarly, the skinhead movement had originated in 1984 under fear of the “foreign threat” (Ausländerbedrohung) [19]. Although “foreigner” seems like a general term, various compounds including Ausländerfeindlichkeit (literally translated as hostility against foreigners) have been taken as clear indicators that Ausländer is not a term which refers to all foreigners (including other white Europeans), but rather to the ethnically or racially diverse [20]. In the late 1990s, measuring immigration and integration programs and limitations had been an important component of German discourse [21], and softer terms such as Migrant (migrant) began to replace Ausländer (foreigner) as a more politically correct alternative [22] – however, the term Ausländer is still widely used.

In the early 2000s, Angela Merkel began her time as German Chancellor and had brought integration more into the forefront of German discourse. Although initially defined as the extent to which social connections are made between groups of people, integration in the German political context was also used as a point of criticism against foreigners, who had allegedly “isolated” themselves, showing no interest in being a part of German society [23]. Merkel´s party, the Christian Democrats (CDU), had established a debate surrounding Leitkultur – the extent to which migrants can and should integrate. Anti-immigrant politics asserted that migrants unwilling to integrate would compromise the cultural integrity of Germany [24–26].

Discourse of the early and mid-2010s took a much harder stance, as Merkel declared the end of multiculturalism in Germany: ‘Multikulti ist tot’ (multiculturalism is dead) – stating that cultural diversity without cultural assimilation threatens peaceful coexistence in Germany [27,28]. Thus, migration in the 20th century had focused on temporary guest workers rather than a true “welcome culture”, and resentful discourse and connotations followed; the 21st century had introduced integration discourse, but under the guise of pessimism.

By 2010, Germany´s foreign population stood at 6.7 million; Germany had the largest foreign population in Europe, which had continued to grow during the 2015 “refugee crisis”, in which Germany had welcomed about 1.2 million refugees from Syria, Iraq, Afghanistan – the largest influx of refugees since the aftermath of World War II [29]. S1 Fig shows the immigration to and from Germany over time. The 2015 refugee crisis marked a new age of discourse for German media, as Merkel swapped Leitkultur for Willkommenskultur (welcome culture), famously exclaiming “Wir schaffen das!” – “we will make it!” rather than “multiculturalism is dead!” [30].

The compounding effects of Germany´s history have resulted in discourse with competing ideologies [31], namely between “welcome culture” and nationalism. There has always been an internal debate as to whether Germany should be considered a “land of immigrants”, including staunch reluctancy to accept foreigners into the country [14]. Success of the populist political party – the Alternative für Deutschland (AfD) – increased notably in the aftermath of the 2015 refugee crisis [32]. As prevalence of the asylum or migration topic rose in media, the AfD had adopted an anti-immigrant platform, which proved itself to be a successful strategy [32]. As the party´s success continues to grow, the topic of immigration in Germany remains one of the most important for party program agendas and media discourse [33]; media´s role in dehumanizing migrants and refugees and its effects has thus been noted [34–36].

**Fig 1:**
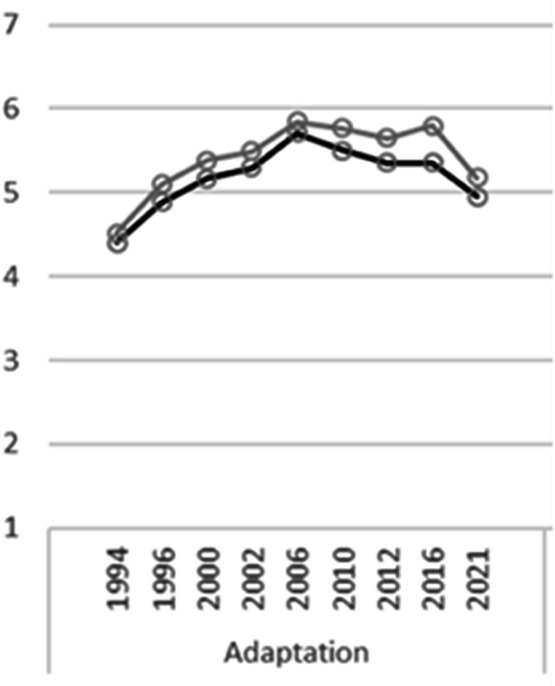
GGSS Item MA01. Foreigners Should Adapt More (Ausländer sollen sich mehr anpassen). Scale of 1 (Strongly Disagree) to 7 (Strongly Agree), 1994-2021. Dark Grey represents West Germany; Light Grey represents East.

A series of events (migration, media reporting, and political adversity) has spurred changes in public opinion. Looking at the German General Social Survey (GGSS), one can see an increase of the demand on the foreigners (GGSS uses term “Ausländer”) – to adapt themselves to the German culture. This demand climbed until 2002, reaching peaks in 2006 and 2016, promptly following the increased number of refugees in the years prior (Fig 1). Despite the controversy of the term “Ausländer”, it is consistently used in social science and public opinion research when studying attitudes towards migrants.

Considering the role mass media has played in depicting social and political events, one point of interest is how media discourse (on themes of immigration and integration) evolves over time. Media discourse has been analyzed in a multitude of ways, including framing [37, e.g.,], the salience of immigration topics in media [38, e.g.,], and the sensationalizing of discourse by right-wing populists [39, e.g.,]. However, the change in “Ausländer” discourse over time has seldom been addressed in the computational sense.

## Theoretical and empirical background

### Immigration and integration

The theoretical framework surrounding immigration and the subsequent process of integration has been established: Social identity theory [40] describes the mechanisms in which people identify with a cohort or group, including the perception of other groups (i.e., Ausländer). The role of political alignment in social identity formation can be observed in Germany´s internal struggle between nationalism and “welcome culture”, in which people identify themselves socially according to political belief [41].

Intergroup threat theory [42] also relates to Germany´s evolving social landscape and the hostile reception of foreigners by natives, particularly over the perceived competition for resources and jobs [43]. Literature supporting threat theory finds that right-wing extremism and prejudice arise by feeling threatened by the “outgroup” (minority group) [44–47], which can be an unfortunate result of “acculturative stress” on the native population [48, p. 378] due to increased foreigner migration into the country. Thus, the connection between political and social identity is made stronger.

Despite the social clashes which arise from mass migration (the perception of threat, the likening to cliques and cohorts), social integration is the result of social participation. Shadid [49, p. 362] further defines social integration as “the participation of ethnic and religious minorities, individually and as groups, in the social structure of the host society [... ]”; whereas Mogahed and Nyiri [50] de-emphasized the cultural conformity aspect of integration and focused on the shared goals and commitment towards the host society. As such, integration itself is a process which requires both consent from the host society, and the willingness of the migrant to integrate. Budyta-Budzyńska [51] describes the spectrum as beginning with segregation (remaining separate from the host population), which leads into adaptation (the gradual and personal change one experiences over time through the acculturation process), which leads to integration (the blending into society more so than before by robust relationships but keeping a distinct national identity), and transforms into the final assimilation, in which the individual or group is similar with the host (in behavior, customs, beliefs, for example). As such, immigration and integration exist as objective events, which are connected to nuanced social theories. The crux is to determine how discourse surrounding these themes change over time, as mass media has been regarded and investigated as a reflection of public discussion [52].

### Agenda setting, salience, and framing

The general assertion is that what becomes a hot topic in the media, becomes a topic in society [53]. Agenda setting includes the media actor’s decision over which stories to cover, how they are framed, including what their intentions are by covering the news through this framing. This access of topics raised by media in the individuals’ minds becomes the state for “issue salience”; the extent to which people cognitively engage with a topic [54].

Goffman [55] had initially theorized that the organization of discourse will affect an individual´s thoughts and actions. As such, framing involves the organization of information. Labeled frames enable people to make necessary interpretations. Minsky [56] defined a frame as a template or data structure that organizes various pieces of information. Gamson and Modigliani [57, p. 143] had explained that frames are the “central organizing idea or storyline that provides meaning”. Over the decades, the intentional negative framing of media, termed a “strategy frame” [58] has gained attention in media discourse research. It has been theorized that negative media bias emerged through press freedom: to criticize politicians and political parties, and political affairs at large [59]. These theories have been applied in literature to enhance media analysis.

## State of the art: Media discourse analysis on immigration

By methodizing the theories, research often aims to determine how salience impacts the reader, how the framing of discourse impacts the audience, or how discourse may shape public opinion. This section provides a summary of the existing literature, depicting the several approaches one may take to analyzing media discourse.

International research on media discourse on immigration includes Hersi [60], who provided informative categorizations about discourse concerning migrants and integration. Hersi claims that the changing discourses of integration could be explained by significant increases of movement of people from one part of the world to another; that literature on integration and immigration discourse has struggled to balance themes of terrorism, radicalization, extremism and integration ever since the terrorist attacks of 9/11 in the USA. However, as Hersi notes, balancing these themes is essential to avoid “othering” specific migrant groups [60]. Hersi´s insight into the nature of discourse balancing is relevant, considering we analyze the various contextual frames of immigration and integration discourse through “Ausländer” terminology.

Also with regard to framing, Räikkönen [61] analyzed in which cases the ingroup is referred to as “we” and “us” and what effects this has on the migrant outgroup for British media reports. Similarly, in the context of the Brexit, when migration to the UK was a salient topic in the media and the society, Islentyeva [62] analyzes how migrants are framed compared to British.

Prior research on media discourse in Germany has focused specifically on the relationship between mass media and the populist radical right party Alternative für Deutschland (AfD) [63–65]. Specifically, Beyer and Weldon [63] used data from German news outlets, Google-trend searches and Twitter, to examine how the AfD party was covered compared to other parties on topics of immigration and Euroscepticism during an election cycle. The authors report that the AfD had a favorable media environment, especially in the final month of the campaign.

Löw and Dewenter [64] examined media landscape bias towards the AfD and showed that mere quantity of times a topic is mentioned in the media can influence how often the public is exposed to it. Their results show that news from several outlets slanted towards AfD topics during the election campaign, ultimately resulting in a biased landscape for political news. However, the authors noted that “the tendency of the reporting is irrelevant, but rather the quantity is decisive” [65, e.g.,]. The disproportionate frequency of coverage for the AfD gives the party more attention, regardless of tone or sentiment. They suggest that there could be an “entertainment factor” which motivates the publication of controversial or negative topics, such as right-wing political parties. These findings attest to the power of high visibility topics and framing are consistent with Koopmans [66], who showed that the visibility of the asylum debate in German media led to an increase in extreme-right violence; it is also consistent to Harteveld *et al*. [67], who found that high visibility of the refugee crisis in the media increases citizens’ Euroscepticism.

Prior attempts at media analysis often attempt to capture visibility of topics or buzzwords as quantifiable phenomenon, potential bias, or whether the media environment pushes a particular agenda; prior literature focuses more so on isolated elements of discourse (for example, specific political parties, politicians or specific sentiments). However, these isolated media elements ignore the polarization of media discourse surrounding migrants, namely between the previously mentioned dichotomy of xenophobia (i.e. anti-immigrant discourse) and welcome culture.

Considering the ongoing battle between nationalist and integration narratives in media discourse – and the historical prevalence and social controversy surrounding “Ausländer”– we aim to uncover discourse categories focusing exclusively on the term “Ausländer”. We address three research questions:

RQ1: What latent discourse categories emerge in German media texts containing ‘Ausländer’ that signal the competing narratives of social integration versus anti-immigrant sentiment?

RQ2: How have the competing “Ausländer” discourse categories changed over the years?

RQ3: What sentiments do these categories have (in particular, is discourse on integration positive)?

We conjecture that discourse categories will reflect this polarized dichotomy between nationalism (anti-immigrant discourse) and welcome culture, we therefore expect a minimum of two categories. Considering the narrative timeline and GGSS data, we expect that anti-immigrant discourse will increase approaching the mid-2010s (presumably because of the critical reception of refugees during/after the unprecedented influx of 2015), but more social integration discourse thereafter. Provided the complicated history of social integration discourse in Germany, we expect Social Integration discourse to have either a neutral or negative sentiment, but not positive.

Therefore, we aim to address broader elements of discourse, including measuring and comparing the prevalence of various frames (categories) in which immigrants are discussed (through a wide spectrum of compound words), and the prevalence of Ausländer-terms within each frame overtime. To take it a step further, we implement a sentiment analysis per category, to better understand the tonality of the discourse as their categorical prevalence changes over time. We take a concrete stance on category semantics: category labels themselves are meant to indicate the discourse topic and not the inherent opinion. Therefore, a category for xenophobic discourse will include text which reports xenophobia in any capacity, including whether the sentiment is positive (critical of xenophobia), negative (supporting anti-immigrant tendencies), or somehow neutral (such as objective statistical information).

## Methodology

### Mass media data collection

We use one GGSS item (originally [MA01]), which we label as “Adaption” as a reference point (Fig 1). This guided our decisions according to which years of media to collect, whether the media data should be national or regional, and serves as further justification as to why we make the term “Ausländer” central to our text analysis. Moreover, the GGSS is administered during key “time frames” of each year, which we adapt as well. Therefore, the media corpora for each year contain articles which correspond specifically to the GGSS survey administration periods. We selected the years 2006, 2010, 2012, 2016, and 2021 and collected our media according to the specific annual windows in which the survey was administered. The combined corpora amount to 10,063 articles. S2 Fig provides a full table describing corpus size per year, media outlets, and time frame for corpus collection.

We used FID Media (formerly called adlr.link) as a resource for our media corpora. It is an archival database provided by the University of Leipzig. FID Media is an academic information service for communication and media studies [68]. Unfortunately, FID Media does not allow for the sharing or republishing of articles, therefore the articles from the corpora can be only accessed directly from the archival database itself; the analysis method itself complied with the terms and conditions of FID Media.

For the term selection strategy, we focused exclusively on the term “Ausländer”. The advantage of this term is that it is rich in etymological history and controversy; despite the stance that it is politically “incorrect”, there are several Ausländer-compound terms available in the German language, which are widely used in media discourse, and serve as the basis for our text analysis. This term is also used in many GGSS items; we would like to maintain the connection between public opinion data and media discourse regarding foreigners by keeping terms consistent; this is especially important as our corpora of published media articles coincides with the survey collection phases. Therefore, our media discourse search was restricted to the keyword “Ausländer” (foreigner) and all articles in the corpus containing this term were selected for the predefined time periods.

### Text cleaning

Once the corpora have been compiled, the next step is to clean the text. *Cleaning the text* typically refers to removing any unnecessary words or symbols from the body of text. Specifically, this involves removing stop words, numbers, punctuation, as well as converting all words to lowercase letters [69].

The cleaning of our corpus was conducted using *nltk* (Natural Language Toolkit), which is a Python package commonly used for removing stop words. A list of 155 stop words were effectively removed from each corpus. Once each corpus was collected, each corpus was then cleaned individually. To identify relevant topics, python libraries *re, defaultdict, Counter* were utilized to return the top 100 most frequently used terms in the corpus. However, the terms “Ausländer” and the conjugation “Ausländern” were excluded as they are not descriptive. In German, compound words are incredibly common, therefore many terms used to describe situations or people are often confounded into one simple term (for example, “Ausländerproblem”, which means foreigner problem, or “Ausländerintegration”, meaning foreigner integration).

NLTK and WordNetLemmatizer were used for the purposes of lemmatizing the terms (combining singular and plural tenses and words of the same meaning). As a result, the frequencies of one term are added to the frequency of another terms (if they are the same in meaning and/or the singular/plural or some conjugated form of the same word, such as Ausländerbehörde/n (a government agency for foreigners) or Ausländerfeind and Ausländerfeindlichkeit (xenophobe and xenophobia). Once the terms for each category (and all their consolidated forms) have been identified and consolidated into their groups, the categories can finally be compared for each year.

### Categorizing media with Latent Semantic Analysis (LSA)

We used Latent Semantic Analysis (LSA) to evaluate the term “Ausländer” in mass media discourse. LSA was particularly suitable, because its categorization is context dependent; semantic “meaning” emerges through the relationships between terms in a context-based subspace [70]. LSA relies mainly on the co-occurrence of terms in order to categorize them [71,72] and operates on the premise that similar words are used in similar contexts. LSA exploits the frequency count to derive a meaning that is purely based on the text (the language itself) [73]. In regard to the lists of consolidated terms, this means that terms which are frequently co-occurring or exist within the same contexts as one another are most likely to be sorted within one category. As terms appear across different categories (as they are discussed in various contexts), overlap in terms across categories is expected.

LSA algorithms commonly perform with td-idf (term frequency—inverse document frequency), which helps LSA to determine the significance of words based on how often (or how infrequently) they appear in the text [74]. It is often used as a tool to reduce noise within a document and can be particularly useful when researchers are attempting to investigate the contents of the document without prior knowledge of its topics. In the current case, although td-idf is an inherent part of the algorithm, its purposes are mediated by the deliberate use of pre-determined terms of interest (the Ausländer compound terms). Furthermore, additional text processing steps –such as the lemmatization of the compound terms – further assisted in the cleaning of text and reduction of noise. In the lemmatization process, terms of the same meaning (for example, ausländerfeind, ausländerfeindlich, and ausländerfeindlichkeit) are combined, along with their frequencies. Therefore, although td-idf is elemental in reducing noise, reducing the analysis to specific terms also effectively reduces noise.

One caveat to LSA is that the number of categories must be predetermined. Therefore, we measured coherence for each corpus to determine what number of categories would be best. For each year (except 2016), three categories showed the highest coherence (with relatively high values ranging from 0.74-0.78). For 2016, four were considered ideal with a score of 0.85. For years 2006, 2010, 2012, and 2021, four categories still achieved satisfactory coherence values, despite that three topics were most recommended. Coherence measurement charts can be found under S3 Fig. Ultimately, we can achieve more nuanced categories with a manageable amount of overlap with four categories. It is important to keep some overlap [75], as it is still a fair indicator of the different contexts in which these terms are used.

Although LSA organizes these terms into respective categories, it does not label the categories themselves. To appropriately label the categories, two coders (Coders 1 and 2) labeled the categorized term cluster for each year. Therefore, the labels should reflect the prevalent terminology within each category (i.e. the category labeled “Xenophobia” has a disproportionately high amount of discourse using the term “Ausländerfeind” whereas the discourse category “Social Integration” more so contains terms such as “Ausländerintegration” or “Ausländerrecht”). The labels therefore concern themselves with the general category theme and is not concerned with the sentiment of the discourse.

The coders received an Excel chart containing four clusters of terms – there was one Excel chart per year, to ensure that each year would have four respectively labeled categories, and to prevent the intermixing of discourse terms from the corpora. The prompt was: “Based on the terms within each group, label the categories as either Administration& Policy Social Integration, Limiting Migration, and Xenophobia. Each group can only have one label, and each category should be represented.”

This framework was intended to reflect a spectrum of discourse, ranging from the bureaucratic (potentially objective), to discourse discussing alternative stances (from pro- to anti-foreigner). The intercoder agreement labels had a Cohen´s K agreement score of 0.64, which is considered substantial [76]. There was a total of twenty clusters (n=20), which are four categories for five years. There was consistent agreement on 14/20 labels. The only disagreement was found between Social Integration and Administration, which is likely due to the nuanced discourse surrounding rights, which can concern bureaucracy and integration alike; moreover, there is a distinction between discourse terms referencing government agencies (such as Ausländerbehörde) and the applicants. In the six instances of disagreement, a third coder (Coder 3) was used to decide and provide rationale.

### Sentiment analysis

Sentiment analysis is a field at the intersection of linguistics and computer science that attempts to determine the sentiment contained in text [77]. It is not purely psychological or emotional in nature, but rather a nuanced approach to how we can classify linguistic expressions according to the type of opinion that they convey [78]. Human beings are inherently emotional beings, which often influences our linguistic expression and interpretation of text, however sentiment analyses are also occasionally a matter of subjective or objective interpretations [77,78]. Therefore, we naturally interpret texts based on fact, our innate emotional reasoning, and opinion. Sentiment analysis, as being intertwined with computer science, has evolved to include non-human analytics. For example, Chat-GPT has been extensively utilized in sentiment analysis for media and social media text [79, e.g.,], including on multilingual texts [80]. Moreover, the comparison between Chat-GPT and human annotation has been extensively explored with the consensus that Chat-GPT provides competitive accuracy [81] across a wide range of text topics, including media text [82]. We adapt this method for our sentiment analysis.

At this point, the number of categories has been established and the categories have been labeled but the discourse within each category still require a sentiment analysis. This step helps us to decipher whether the discourse in a given category (i.e. Social Integration) contains positive, negative, or neutral discourse. The sentiment analysis provides valuable insight into each category, as (for example) it may be the case the category “Xenophobia” contains discourse against Xenophobia, but it nonetheless categorized with “Xenophobia” discourse for the terminology provided and the fundamental topic being discussed. Therefore, it could be that an increase in categorical prevalence for Xenophobia does not necessarily mean growing disdain for migrants in media. For this reason, a sentiment analysis is in order. Given the bank of prior literature using Chat-GPT for inter-reliability, we compare the Chat-GPT annotations to human (German native speaker). Both Chat-GPT (Coder 1) and the human coder (Coder 2) were provided sentences (n=296); markers such as year and category labels were removed to prevent bias. Coders were given the same prompt: “Based on the “Ausländer” compound terms, please label each sentence as either positive, negative, or neutral in sentiment.”A category which achieves the majority consensus of any sentiment is deemed to be positive, negative, or neutral overall (i.e., 50% positive, 25% negative, 25% neutral). We calculated the agreement of human annotation and Chat-GPT with Cohen´s Kappa, which is a robust measurement of agreement that accounts for degrees of randomness in the sample and follows this scale: ≤ 0 indicates no agreement, 0.01–0.20 as none to slight, 0.21–0.40 as fair, 0.41– 0.60 as moderate, 0.61–0.80 as substantial, and 0.81–1.00 as almost perfect agreement [83]. Confidence intervals are also reported with the results for each sentiment analysis per category.

## Results

### Categories extracted

Addressing RQ1: Given the competing “pro- and anti-immigrant” themes in media discourse, we had hypothesized a minimum of two categories. When using the term “foreigner” (Ausländer) as a reference, we found the following four categories that described the media discourse when using the term Ausländer as a reference:

**Administration and Policy** emphasizes government agencies or contains bureaucratic terms. This category was labeled for the following terms having highest weights: Ausländerbehörde and Ausländerbeirat, which are organizations that typically assist foreigners in a wide spectrum of tasks (applying for residency permits, visas, asylum, etc.). Therefore, this context represents the legalities surrounding migration. One example from this, direct from the corpus (translated from German) is: “Now, a politically controversial question that has long been a hot topic of debate is being passionately debated again: Are we deporting the wrong people? And is what our immigration authorities (Ausländerbehörden) are doing right – is it humane?” (Die Zeit, 2021). Another such context of administrative terminology includes: “In response to a BamS (Agency for Migration Services) inquiry, the responsible immigration office (Ausländerbehörde) explained that [a] “family reunification visa” for permanent residence in Germany [... ]” (Frankfurter Allgemeine, 2021).

**Limiting Migration** includes anti-immigrant discourse combined with policy discourse. It was so named for the weighted emphasis on Ausländerbeschränkung (to limit foreigners), Ausländerfeind (xenophobia), or “Ausländerkriminalität” (to the effect of foreigner criminality). One example direct from the corpus (translated from German) is: “Plans to restrict foreign players (Ausländerbeschränkung) and increase playing time for German talents in the league should be implemented quickly. Now seems like the right time” (Die Welt, 2010). Another example being: “Foreigner criminality (Ausländerkriminalität) and Islam is not a winning theme in the Union” (Bild, 2021). The intermingling of anti-immigrant discourse with legal contexts causes a lot of nuances, which on the one hand enhances our analysis by uncovering variety among a dichotomy of polarized discourse. On the other hand, it can complicate the analysis (for instance, in the case of coherence values straddling between three and four topics; moreover, the nuance can also be construed as overlap).

**Xenophobic** discourse has some overlap with “Limiting Migration” however contained less content surrounding policy and has more emphasis on the anti-immigrant sentiment alone, including terms such as: Ausländerhass (hatred of foreigners), Ausländerproblem (the foreigner problem), Ausländerfeindlichkeit (xenophobia), etc. Therefore, discourse which use these terms categorically discuss themes of xenophobia, which includes discourse which may include both discourse which discusses anti-immigrant sentiment, and also discourse which is critical of xenophobia, but is nonetheless discussing xenophobia as a topic of discourse. One example is as follows: “Following a xenophobic attack on a Turkish woman in Giesing, police are seeking witnesses whose information could help in the search for the perpetrator” (Süddeutsche Zeitung, 2006). Another example includes: “So far, the only party I can really rule out is the AfD. They’re a xenophobic party. What they’re doing isn’t right.” (Die Welt, 2021).

**Social Integration** contained discourse including terms Ausländerintergration (integration of foreigners), Ausländerhilfe (helping foreigners), Ausländerrecht (law to regulate foreigner rights), and Ausländerwahlrecht (the right to vote for foreigners). It should be noted that Social Integration should be construed objectively; despite that the discourse pertains to the inclusion of foreigners into society, it cannot and should not be assumed that the discourse itself is “pro-immigrant” per se, but in any case, a discussion about integration. This discourse can be categorized as integration issue in accordance with Shadids’ [49] participation focus. One translated example is: ““We will fight for the right to vote,” said Raed Saleh. The group is open not only to SPD members, but also to fellow activists from other political camps and from associations of foreign communities. Mohamad Beidoun from the German-Arab umbrella organization supports the initiative” (Die Welt, 2010). Another example of context in which terms related to social integration is discussed includes this excerpt: “It’s about two splinter parties that only entered the Römer because there is no five percent threshold in Frankfurt: on the one hand, Yildiz’s BIG party, which advocates for foreigners’ voting rights and for people with a migration background to retain their cultural identity [... ]” (Die Zeit, 2021).

### Change in discourse

**Fig 2:**
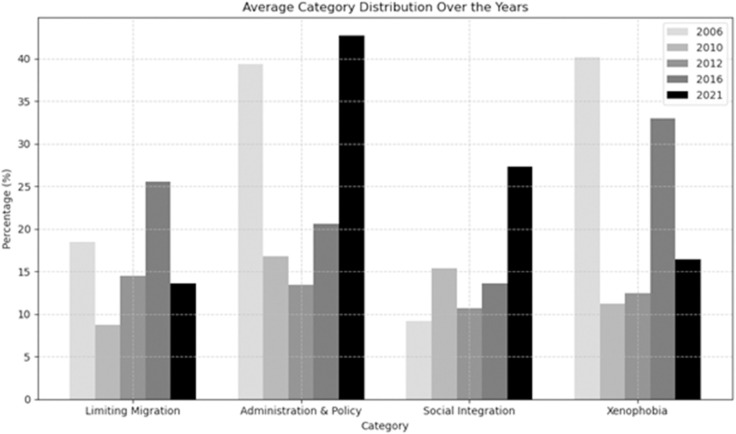
Categorical comparison over time.

Given the previous overview of what each category represents, we now address RQ2 with Fig 2, which represents the side-by-side categorical comparison for all four categories for 2006, 2010, 2012, 2016, and 2021. This ultimately shows the average prevalence of each category per year, based on the categorized terms from each unique corpus. These results effectively compare the discourse of each category relative to the other categories. ANOVA and Tukey HSD analyses were used to determine whether the discourse changes significantly for each individual category by conducting pairwise comparison of the years (each category was the dependent variable and the year was independent variable). Results are described for each category below.

### Limiting migration

The ANOVA results in Table 1 indicate that there are significant differences in attitudes toward Limiting Migration across the years examined (F(4, 70) = 3.72, p = 0.008), suggesting that public opinion or policy emphasis has shifted over time. As shown in Table 2, post-hoc analysis using Tukey’s HSD test reveals that 2012, 2016, and 2021 all differ significantly from 2006, with each showing a substantial change in limiting migration discourse (p < 0.05). However, no significant differences were found between the more recent years (2012–2021), indicating that the changes largely occurred between 2006 and 2012, and then stabilized afterward. Compellingly, the discourse on Limiting Migration was most prevalent during the more “conservative” years of opinion (such as 2006 and 2016), according to our GGSS item on adaptation attitudes Fig 1. Although a causal link between this discourse category and change of opinion cannot be directly made, the results show a correlation between a rise in this discourse and a more conservative opinion at corresponding points of time.

**Table 1:** ANOVA for limiting migration across years.

Source	SS	df	MS	F	p	Effect Size ($\eta^{2})$	$\omega^{2}$
*Year*	4580.45	4	1145.11	3.72	.008	0.175	.12
*Residual*	21550.53	70	307.86				
*Total*	26130.99	74					

**Table 2:** Tukey HSD Post-Hoc comparisons for limiting migration across years.

Comparison	Mean Diff.	p-value	95% CI Lower	95% CI Upper	Sig.
2006*vs*.2010	–9.60	0.567	–27.54	8.34	No
2006*vs*.2012	–19.00	0.033	–36.94	–1.06	Yes
2006*vs*.2016	–19.67	0.025	–37.61	–1.73	Yes
2006*vs*.2021	–19.80	0.023	–37.74	–1.86	Yes
2010*vs*.2012	–9.40	0.587	–27.34	8.54	No
2010*vs*.2016	–10.07	0.521	–28.01	7.87	No
2010*vs*.2021	–10.20	0.508	–28.14	7.74	No
2012*vs*.2016	–0.67	1.000	–18.61	17.27	No
2012*vs*.2021	–0.80	1.000	–18.74	17.14	No
2016*vs*.2021	–0.13	1.000	-18.07	17.81	No

Note. A significance level of p < .05 was used to determine statistically significant differences. Effect size ($\eta^{2}$) reflects the proportion of variance explained by the model. Significant results are marked as ‘Yes’.

Considering Germany had experienced its largest mass-migration in 2015, it perhaps makes sense that “limiting migration” discourse is most prevalent in 2016 (compared to 2006 and 2021). The relationship between Xenophobic discourse and Limiting Migration is important to consider: Whereas 2006 held the most Xenophobic discourse within its own category, it was 2016 which had the most discourse on “limiting migration”, however the difference in discourse in the surrounding years does not appear to be statistically significant. Historically, the lowest number of immigrants since reunification was recorded in 2005 — the number fell further in 2006 [84].

### Administration & policy

As shown in Table 3, ANOVA indicated a statistically significant difference in Administration & Policy scores across years, F(4, 45) = 3.44, p = .016. Post-hoc comparisons using Tukey’s HSD test, as shown in Table 4, revealed that 2006 had significantly higher scores than 2012, 2016, and 2021, suggesting a decrease in policy administration-related measures over time. No other comparisons between years reached statistical significance. This is consistent with Fig 2, which shows Administrative discourse is more prevalent in 2006 than in other years. One potential explanation for the lack of prevalence in 2016 relative to other years is that the discourse surrounding the “refugee crisis” of 2016 placed more emphasis on the critical social reception rather than the administrative aspects of this. In 2006, Administration & Policy constitutes 39.4% of the discourse using “Ausländer”, almost as much as Xenophobic discourse. For 2021, most of the discourse was Administrative in nature (42.7%).

**Table 3:** ANOVA for administration & policy across years.

Source	SS	df	MS	F	p	Effect Size ($\eta^{2})$	$\omega^{2}$
*Year*	21807.32	4	5451.83	3.44	0.16	0.234	.18
*Residual*	71355.90	45	1585.69				
*Total*	93163.22	49					

**Table 4:** Tukey HSD Post-Hoc comparisons for administration & policy across years.

Comparison	Mean Diff.	p-value	95% CI Lower	95% CI Upper	Sig.
2006*vs*.2010	–32.80	0.363	–83.40	17.80	No
2006*vs*.2012	–51.40	0.045	–102.00	–0.80	Yes
2006*vs*.2016	–53.90	0.032	–104.50	–3.30	Yes
2006*vs*.2021	–54.70	0.028	–105.30	–4.10	Yes
2010*vs*.2012	–18.60	0.833	–69.20	32.00	No
2010*vs*.2016	–21.10	0.760	–71.70	29.50	No
2010*vs*.2021	–21.90	0.760	–72.50	28.70	No
2012*vs*.2016	–2.50	1.000	–53.10	48.10	No
2012*vs*.2021	–3.30	1.000	–53.90	47.30	No
2016*vs*.2021	–0.80	1.000	–51.40	49.80	No

### Social integration

As shown in Table 5, ANOVA for Social Integration revealed a statistically significant difference in discourse across years, F(4, 55) = 6.61, p < .001. Post-hoc comparisons using Tukey’s HSD test showed that 2021 significantly differed from all other years (2006, 2010, 2012, 2016), with higher mean levels of social integration, as shown in Table 6. No significant differences were found among the years prior to 2021, suggesting a marked increase in social integration specifically in that year. This is consistent with Fig 2, which depicts 2021 being something of a “hallmark year” for social integration discourse. Considering what is known about integration discourse and “Willkommenskultur” in Germany in during the 2015 refugee crisis and the time immediately thereafter, it is perhaps surprising that the years prior to 2021 were not statistically significant. This could be due to a lack of GGSS data availability (as there was no surveyed year between 2016 and 2021), and/or due to the complicated and jaded history Germany has with integration discourse.

**Table 5:** ANOVA for social integration across years.

Source	SS	df	MS	F	p	Effect Size ($\eta^{2})$	$\omega^{2}$
*Year*	3626.57	4	906.64	6.61	0.002	0.33	.28
*Residual*	7540.48	55	137.10				
*Total*	11167.05	59					

**Table 6:** Tukey HSD Post-Hoc comparisons for social integration across years.

Comparison	Mean Diff.	p-value	95% CI Lower	95% CI Upper	Sig.
2006*vs*.2010	–0.42	1.000	–13.90	13.07	No
2006*vs*.2012	–0.54	1.000	–14.02	12.94	No
2006*vs*.2016	–1.33	0.999	–14.82	12.15	No
2006*vs*.2021	18.83	0.002	5.35	32.32	Yes
2010*vs*.2012	–0.13	1.000	–13.61	13.36	No
2010*vs*.2016	–0.92	1.000	–14.40	12.56	No
2010*vs*.2021	19.25	0.002	5.77	32.73	Yes
2012*vs*.2016	–0.79	1.000	–14.27	12.69	No
2012*vs*.2021	19.38	0.002	5.89	32.86	Yes
2016*vs*.2021	20.17	<.001	6.69	33.65	Yes

### Xenophobic discourse

As shown in Table 7, ANOVA showed a statistically significant effect of year on xenophobia scores, F(4, 100) = 4.36, p = .003, indicating that levels of xenophobic discourse varied across the sampled years. Post-hoc Tukey HSD comparisons revealed, as shown in Table 8 that 2016 differed significantly from 2010, 2012, and 2021. Specifically, xenophobic discourse was significantly higher in 2016 than in both 2010 and 2012, and significantly lower in 2021 compared to 2016. No other year pairs showed significant differences.This fits to Fig 2, in which Xenophobia was most prevalent in 2016 relative to other categories. Compellingly, discourse on Xenophobia was most prevalent during the most “conservative” years of opinion on our GGSS item (also 2016), which draws another interesting parallel between media discourse and opinion trends from corresponding points of time.

**Table 7:** ANOVA for Xenophobia across years.

Source	SS	df	MS	F	p	Effect Size ($\eta^{2})$	$\omega^{2}$
*Year*	3396.34	4	849.09	4.36	.003	0.15	.11
*Residual*	19473.71	100	194.74				
*Total*	22870.05	104					

**Table 8:** Tukey HSD Post-Hoc comparisons for Xenophobia across years.

Comparison	Mean Diff.	p-value	95% CI Lower	95% CI Upper	Sig.
2006*vs*.2010	–5.19	0.748	–17.15	6.77	No
2006*vs*.2012	–4.76	0.803	–16.73	7.20	No
2006*vs*.2016	9.14	0.219	–2.82	21.11	No
2006*vs*.2021	–6.19	0.605	–18.15	5.77	No
2010*vs*.2012	0.43	1.000	–11.54	12.39	No
2010*vs*.2016	14.33	0.011	2.37	26.30	Yes
2010*vs*.2021	–1.00	0.999	–12.96	10.96	No
2012*vs*.2016	13.90	0.014	1.94	25.87	Yes
2012*vs*.2021	–1.43	0.997	–13.39	10.54	No
2016*vs*.2021	–15.33	0.005	–27.30	–3.37	Yes

### Sentiment analysis results

In regard to RQ3, the sentiment analysis revealed that none of the four categories were positive. Although “Social Integration” may imply a positive shift in societal acceptance, the sentiment analysis results reveal that the discourse is neutral at best – more neutral than any other category. This fits our hypothesis, in which we assumed based on the historical pessimism surrounding integration discourse, that this category would also not be positive in sentiment. In fact, there was a practically equal amount of neutral and negative sentiment. A set of 59 consensus-labeled sentences (the sentences which yielded total agreement between coders) indicated that 40.7% of sentences were coded as neutral (95% CI = 29.1% – 53.8%), 37.3% as negative (95% CI = 25.7% – 50.5%), and 22.0% as positive (95% CI = 12.9% – 35.7%).

The negative sentences primarily highlighted challenges or shortcomings in integration policies, whereas positive sentences emphasized efforts to improve integration, and promote inclusion, particularly for families and children. Neutral sentences often provided descriptive or legalistic information without evaluative judgment. The confidence intervals reflect the uncertainty in these estimates due to the modest sample size of sentences analyzed: the challenge here is twofold, as social integration discourse (as shown in Fig 2), was not so prevalent prior to 2021, therefore sample sentences are unevenly distributed across the years. Moreover, (as previously stated), we collected the corpora according to GGSS´s data collection periods: year 2021 had the smallest collection frame, which means that the 2021 corpus was also the smallest. These limitations influence corpus size, and therefore sentence sampling for the sentiment analysis.

Nevertheless, the neutral (borderline negative) sentiment of Social Integration is consistent with prior literature which had established social integration discourse in Germany as being latent with criticism towards foreigners, rather than an optimistic (or positive) welcome culture. Additionally, this category achieved the most intercoder agreement of all four categories, with a Cohen´s Kappa score of 0.642. Table 9 demonstrates the percentage of agreement for each category.

**Table 9:** Cohen’s Kappa and intercoder agreement.

Social Integration	Xenophobia	Limiting Migration	Administration
Neutral 54.84%	Negative 55%	Negative 48.61%	Negative 53%
Cohen´s 0.642	Cohen´s 0.61	Cohen´s 0.41	Cohen´s 0.485

Sentiment and Cohen´s Agreement for each category.

By stark contrast, the most *disagreement* was found on the category Limiting Migration (0.41). The most likely culprit of this disagreement is: of all four categories, Limiting Migration had the most “interdisciplinary” discourse; it contained bureaucratic or policy-oriented discourse on limiting migration, (including crimes committed by foreigners and border control), which is easily construed as anti-immigrant. If we had used three categories rather than four, it is likely that there would have been higher intercoder agreement. Most sentences conveyed negative sentiment toward migrants or migration-related issues (17 out of 36, 47%). Positive sentiment, emphasizing legal fairness, integration, or recognition of migrant contributions, was less frequent (9 sentences, 25%). Neutral or informational statements accounted for the remaining sentences (10 sentences, 28%).

This pattern suggests that discourse in this category is predominantly critical, reflecting concerns over migration restrictions, social tensions, or enforcement policies, while only a minority of texts highlight positive or neutral perspectives on migration management. This also affirms the appropriate label assigned to the category based on the most prevalent terminology that had been clustered with LSA.

Unsurprisingly, the category Xenophobia contained predominantly negative discourse with a Cohen´s agreement of 0.61, which is considered a sufficient degree of agreement. It had the second-highest agreement (after Social Integration). This supports prior literature which had reported on the use of key “Ausländer” terms (Ausländerfeind in particular) as being historically negative in connotation, although not exclusively so. In the Xenophobia discourse, around 59% of sentences are negative, 26% positive, and 15% neutral. These proportions are estimated with 95% confidence intervals of 0.49–0.68 (negative), 0.17–0.37 (positive), and 0.09–0.25 (neutral), indicating that the discourse is primarily negative, with some positive content highlighting anti-xenophobia efforts, and relatively few neutral statements. It is interesting to note that the Xenophobia category contains more “positive” discourse than the Social Integration discourse, presumably because the category discussing Xenophobia includes discourse combating Xenophobia. It is important to see the distinction between discourse which is critical of Xenophobia and pro-integration discourse: Discourse which discusses Xenophobia in a critical way is often addressing a problem with intolerant German natives, which is entirely different from a discussion surrounding the migrant population.

Regarding Administration and Policy – both human and Chat-GPT coders found a negative bias in this discourse: Cohen´s agreement for the sentiment analysis was 0.485, which is considered moderate. In this category, sentiment analysis of 62 consensus-labeled sentences revealed a predominance of negative sentiment (57.4%, 95% CI = 44.2% – 69.7%), followed by neutral (26.2%, 95% CI = 15.8% – 38.9%) and positive sentences (18.0%, 95% CI = 9.4% – 30.0%). Negative sentences highlighted bureaucratic obstacles, restrictive policies, and challenges faced by immigrants when interacting with local or federal authorities. Positive sentences focused on administrative initiatives, support programs, and constructive actions by Ausländerbeauftragte or advisory boards. Neutral sentences largely contained factual or statistical information regarding registries, legal provisions, or the role of administrative actors. The confidence intervals reflect some uncertainty due to the sample size of sentences analyzed.

Therefore, the sentiment analysis results suggest that it was easier and more reliable to label Social Integration and Xenophobic discourse. One potential reason for the higher agreement towards “integration” discourse is that matters of anti-immigrant are more nuanced in nature: discourse may be critical of migration politics but not anti-immigrant, per se. Each category (except Social Integration) was found to be predominantly negative, with varying degrees of agreement.

### Summary of results and discussion

In response to our first research question: we found four main topics as frames of the media discourse with a reference to the overacting topic “Ausländer”. However, these theoretically could have been reduced to three. Our second research question addresses the change of discourse over time: The media discourse trend shows that the prevalence of xenophobic discourse spiked in 2006 and again in 2016. In regard to the categories Xenophobia and Limiting Migration, our hypothesis was correct, although the difference between discourse was significant for Xenophobic discourse relative to other years of discourse within that category, whereas 2016 Limiting Migration discourse did increase, the difference relative to other years in that category were not significant. The hypothesis expecting anti-immigrant discourse to rise in the mid 2010s was therefore correct, albeit with caveats. The second half of the hypothesis (pertaining to social integration) was accurate, in that social integration discourse did increase after mid 2010s; despite that integration discourse had been slowly rising over the years from a descriptive standpoint, only the year remains significantly different from the others. Lastly, in regard to our third question regarding sentiment: Results reflect a negative sentiment for all discourse categories except Social Integration, which was mostly neutral (by a narrow margin). However, results show that Social Integration discourse is still more negative than positive, upholding the theoretically jaded framework surrounding Germany´s “welcome culture”. In this regard, our hypothesis based on integration skepticism was accurate: integration discourse is not inherently positive, despite the increase over time. The sentiment analysis for Social Integration had the highest agreement, followed by Xenophobia. The polarized discourse is likely harder to define, whereas Administration and Limiting Migration had lower agreement. Potential options for improving agreement include dichotomizing the sentiment (Positive and Negative, no Neutral), testing if agreement is better between two native speakers (as opposed to one native speaker and ChatGPT). Another option would be to introduce alternative forms of sentiment analysis, such as a pre-trained German lexicon approach. In addition to the triangulation of results, here are a number of limitations/challenges to consider:

1. Our analysis was restricted to the term “foreigner”. Therefore, these results only attest to how discourse pertaining to foreigner compound buzzwords has changed over time. Although the term “Ausländer” (foreigner) has historical relevant and controversy, the term become antiquated(and politically incorrect); alternative terms such as migrant and immigrant may have grown in prevalence. Therefore, it is of crucial interest to extend this analysis, to compare the prevalence and usage other terms, and to understand the contexts in which these alternative terms are used. Hence, as the term “foreigner” is a relevant reference point in the German language and can be used to examine the etymology of synonyms (such as “migrant” or “immigrant”).
2. The success of this application on our corpus may be largely due to the nature of the German language itself; the German language uses and creates compound words so often, which allowed us to streamline the search for discourse on foreigners, and terms describing foreigners (Ausländer compound terms). This is not so much a limitation for the current analysis but poses a challenge to implement this method with text in other languages (such as English).
3. It would be remiss to not discuss the differences between prevalence and salience. The present paper is concerned with the former, as we cannot attest to the effects of these terms on media consumers (readers). However, we can and do attest to the changes in prevalence of key words and categorically overall.

## Establishing results in existing framework

Our research extends the text analysis of Mazzoleni *et al*. [65] by showing the frame of the discussion on migration and its change over time. While some previous media analyses focuses on the polarization on social integration discourse [85], we can show a relative prevalence of this topic among the other relevant discourse categories. In addition, our results do demonstrate a polarization within the social integration discourse, which is assigned to other topics such as xenophobia.

However, it should be noted that an increase in Social Integration discourse does not mean that right-wing media is denounced or that the presence is less prominent, especially considering the neutral (borderline negative) sentiment surrounding integration. Therefore, our results do not contradict the results from Beyer and Weldon [63] and similar research, attesting to favorable media landscapes for AfD and anti-immigrant salience. Their results are rather in line with our finding of a high prevalence of xenophobic and limiting migration discourse in the media throughout the years. Further research should compare the landscape among pro-immigration and integration terminology with terminology surrounding right-wing populism (and the anti-immigrant agenda). Our results also somewhat support literature which attests to negative media bias or entertainment bias (e.g., hypothesized by Löw and Dewenter [64]. To this end, additional text analysis for various terms (including migrants, immigrants), should be investigated, and computational applications should be leveraged to investigate negative media bias further

## Supporting information

S1 Fig Migration to and from Germany (1991-2020). (DOCX)

S2 Fig Corpora Collection (2006, 2010, 2012, 2016, 2021). Corpus size per year, media outlets, and time frame for corpus collection.
(DOCX)

S3 Fig Coherence Values (2006-2021 Corpora). Each corpus recommends three categories, 2021 recommends four.
(DOCX)
