# Supplementary figures and images for: Foreigners welcome? Categorizing change in German mass media discourse with Latent Semantic Analysis (LSA)
Source: PLoS One. 2026 Feb 13;21(2):e0340164. doi: 10.1371/journal.pone.0340164 (PMC12904583; doi:10.1371/journal.pone.0340164)

**S1 Fig. Immigration to and from Germany (1991 -2020).**
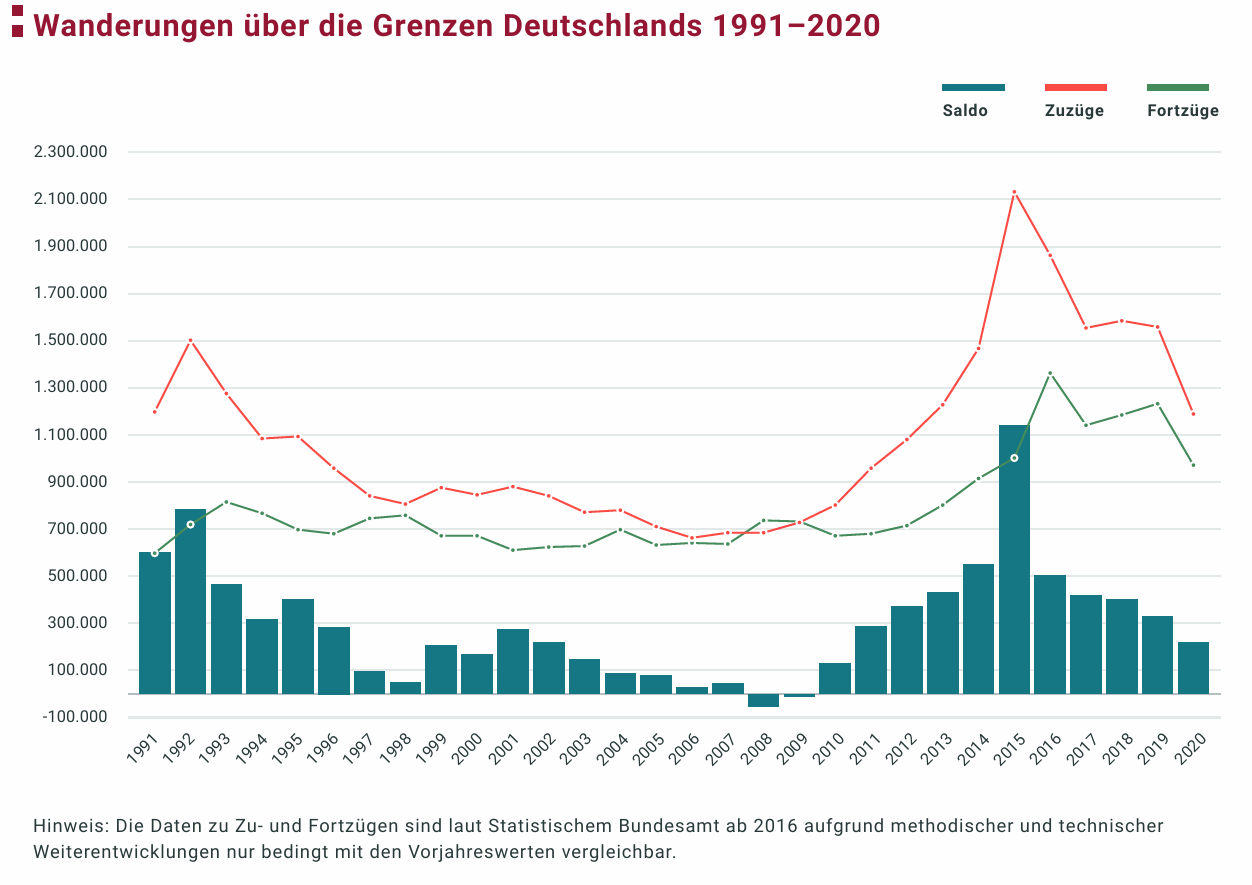
*S1*

Supplement: S1 Fig — (DOCX) [file pone.0340164.s001.docx]

**S3 Fig. Coherence Values (2006-2021 Corpora).** Each corpus recommends three categories, 2021 recommends four.

**
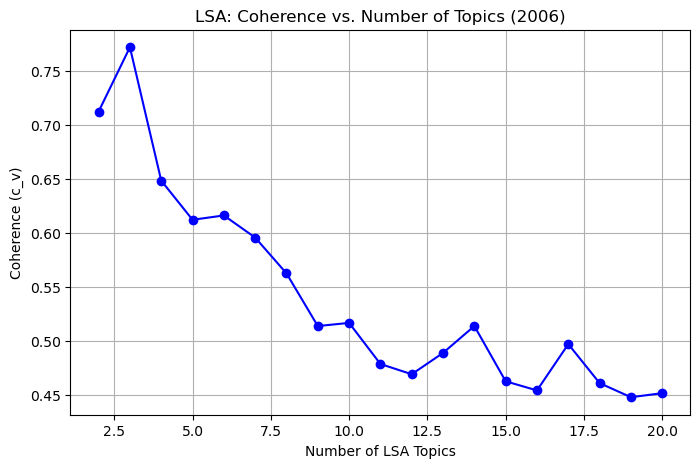
**

**
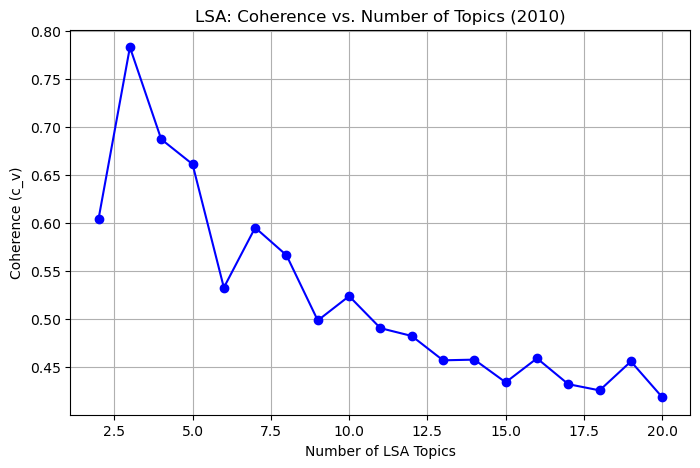
**

**
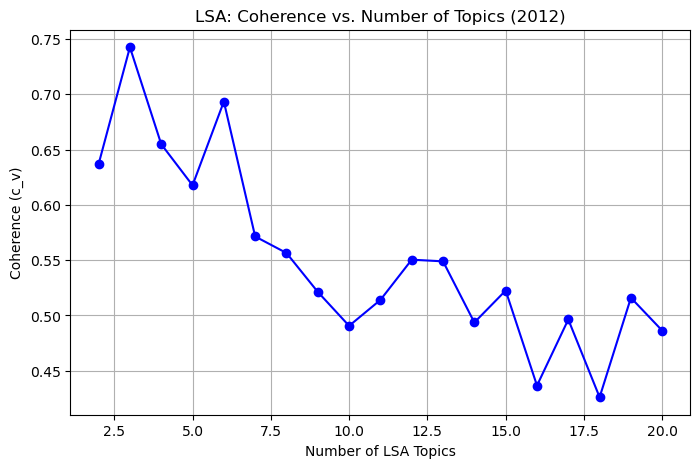
**

**
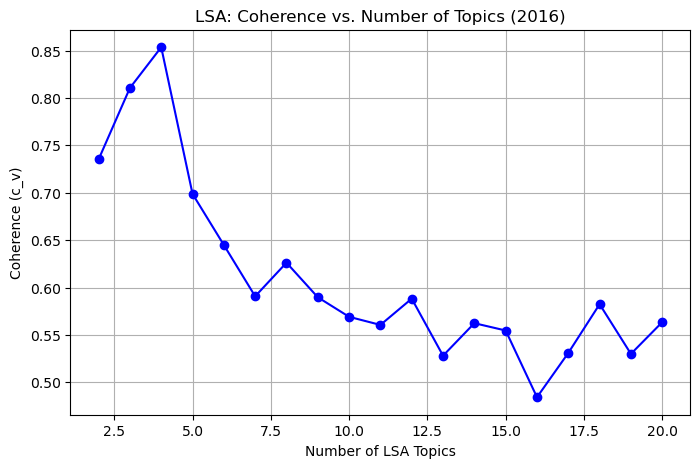
**

**
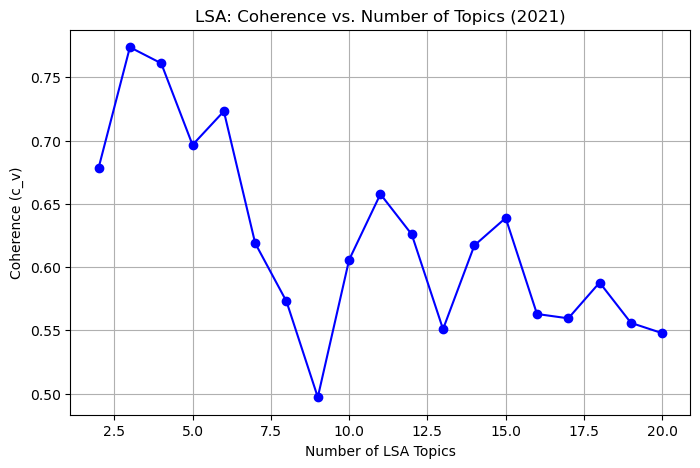
**

Supplement: S3 Fig — Each corpus recommends three categories, 2021 recommends four. (DOCX) [file pone.0340164.s003.docx]
