# Supplementary material for: Foreigners welcome? Categorizing change in German mass media discourse with Latent Semantic Analysis (LSA)
Source: PLoS One. 2026 Feb 13;21(2):e0340164. doi: 10.1371/journal.pone.0340164 (PMC12904583; doi:10.1371/journal.pone.0340164)
Supplement: S2 Fig — Corpus size per year, media outlets, and time frame for corpus collection. (DOCX) [file pone.0340164.s002.docx]

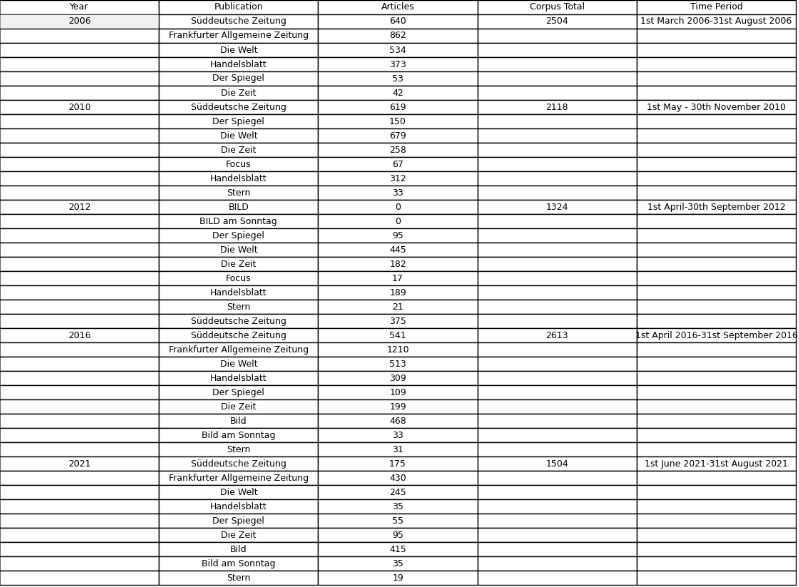
**S2 Fig. Corpora Collection (2006, 2010, 2012, 2016, 2021).** Corpus size per year, media outlets, and time frame for corpus collection
